# HMGB in Mollusk *Crassostrea ariakensis* Gould: Structure, Pro-Inflammatory Cytokine Function Characterization and Anti-Infection Role of Its Antibody

**DOI:** 10.1371/journal.pone.0050789

**Published:** 2012-11-29

**Authors:** Ting Xu, Shoubao Yang, Jiasong Xie, Shigen Ye, Ming Luo, Zewen Zhu, Xinzhong Wu

**Affiliations:** 1 Laboratory of Marine Life Science and Technology, College of Animal Sciences, Zhejiang University, Hangzhou, Zhejiang, China; 2 School of Life Sciences, Shaoxing University, Shaoxing, Zhejiang, China; 3 College of Life Science and Biotechnology, Dalian Ocean University, Dalian, Liaoning, China; University Medical Center Utrecht, The Netherlands

## Abstract

**Background:**

*Crassostrea ariakensis* Gould is a representative bivalve species and an economically important oyster in China, but suffers severe mortalities in recent years that are caused by rickettsia-like organism (RLO). Prevention and control of this disease is a priority for the development of oyster aquaculture. It has been proven that mammalian HMGB (high mobility group box) can be released extracellularly and acts as an important pro-inflammatory cytokine and late mediator of inflammatory reactions. In vertebrates, HMGB’s antibody (anti-HMGB) has been shown to confer significant protection against certain local and systemic inflammatory diseases. Therefore, we investigated the functions of Ca-HMGB (oyster HMGB) and anti-CaHMGB (Ca-HMGB’s antibody) in oyster RLO/LPS (RLO or LPS)-induced disease or inflammation.

**Methodology/Principal Findings:**

Sequencing analysis revealed Ca-HMGB shares conserved structures with mammalians. Tissue-specific expression indicates that Ca-HMGB has higher relative expression in hemocytes. Significant continuous up-regulation of Ca-HMGB was detected when the hemocytes were stimulated with RLO/LPS. Recombinant Ca-HMGB protein significantly up-regulated the expression levels of some cytokines. Indirect immunofluorescence study revealed that Ca-HMGB localized both in the hemocyte nucleus and cytoplasm before RLO challenge, but mainly in the cytoplasm 12 h after challenge. Western blot analysis demonstrated Ca-HMGB was released extracellularly 4–12 h after RLO challenge. Anti-CaHMGB was added to the RLO/LPS-challenged hemocyte monolayer and real-time RT-PCR showed that administration of anti-CaHMGB dramatically reduced the rate of RLO/LPS-induced up-regulation of LITAF at 4–12 h after treatment. Flow cytometry analysis indicated that administration of anti-CaHMGB reduced RLO/LPS-induced hemocyte apoptosis and necrosis rates.

**Conclusions/Significance:**

Ca-HMGB can be released extracellularly and its subcellular localization varies when stimulated with RLO. Ca-HMGB is involved in oyster immune reactions and functions as a pro-inflammatory cytokine. Anti-CaHMGB can significantly suppress RLO/LPS-induced inflammatory responses and hemocyte necrosis and apoptosis, suggesting that Ca-HMGB is a potential target to prevent and control RLO/LPS-induced disease or inflammation.

## Introduction

Mollusca is one of the most diverse groups of invertebrates with more than 100,000 living species, mostly located in marine environments [1]. Recently, there are increasing numbers of studies carried out on comparative immunology using molluscan models because this prominent invertebrate phylum contains a goldmine of information with relevance to immune evolution and provides a comprehensive view of innate immunity across the broad spectrum of invertebrate phyla. These recent studies updated our general understanding of invertebrate immune systems from a simple and homogeneous system to a more sophisticated and diversified immune system capable of defending against pathogens, which include more than the production of limited repertoires of traditional pattern recognition molecules [2]–[16]. The oyster *Crassostrea ariakensis* Gould is one of the representative species of bivalve and it is also one of the most economically important oysters cultivated in southeastern China, such as in Guangxi, Guangdong and Fujian provinces. The oyster cultivating industry significantly contributes to these provinces’ financial revenue and plays an important role in the lives of local people. However in recent years, oyster culture has suffered from severe mortality caused by the pathogen rickettsia-like organism (RLO) [17], [18]. Rickettsias are Gram-negative bacteria, generally described as obligate intracellular pathogens that multiply only within host cells [19]. This prokaryote has been reported in many aquatic species including fishes [20], [21], crustaceans [22], [23] and mollusks [24], [25]. In marine mollusk, more than 25 species in the world have been reported to be infected with RLOs [25], resulting in mollusk mortalities and dramatic economic losses since the first report by Harshbarger et al. (1977) in *Mercenaria mercenaria* [26]. Now prevention and control of this disease is becoming the priority for the development of oyster aquaculture. In the past years, we have made significant progress in collecting data regarding the oyster defense system [11], [12], [27], [28], especially from the hemocyte cDNA library we constructed [29]. Here, we report a novel high mobility group box (HMGB) protein from the mollusk *Crassostrea ariakensis* Gould, which we named Ca-HMGB.

**Table 1:** Sequences of primer pairs used in this study.

Putative Gene	Primer Sequence(5′–3′)
**HMGB ORF primer F**	TT***GGATCC***ATGGGAAGAAAGGACGGA
**HMGB ORF primer R**	GG***CTCGAG***TTACTCGTCATCATCTTC
**HMGB real-time RT-PCR primer F**	AGAAGAGAGCAAAGGACCCA
**HMGB real-time RT-PCR primer R**	TGGTAACCTTCTCCCACCTC
**28s real-time RT-PCR primer F**	GAATCCCTCATCCTAGCGA
**28s real-time RT-PCR primer R**	CACGTACTCTTGAACTCTCTC
**LITAF real-time RT-PCR primer F**	GAGAAGTCAGGACCCCA
**LITAF real-time RT-PCR primer R**	TTAGATTCTGTCGTAGCG
**Myd88 real-time RT-PCR primer R**	CAGGAGTTCGTCAGTCTC
**Myd88 real-time RT-PCR primer F**	GACTCTCAGCTCTTTCTTG
**TGFβ real-time RT-PCR primer R**	GAGAATAGTGGTCTGGTGAT
**TGFβ real-time RT-PCR primer F**	CAGGCTGCATGAACGACTG
**AIF1 real-time RT-PCR primer R**	GGCAAAGACCCATCTAGAGC
**AIF1 real-time RT-PCR primer F**	CTGCGGGTTTAGCTTTCTCT

Restriction enzyme sequences were in bold and italic.

HMGB protein is an abundant, non-histone chromosomal protein and highly conserved in all eukaryotes [30]. Historically, HMGB was known as a nuclear DNA-binding protein and functions as an architectural element that modifies the structure of DNA and chromatin to generate a conformation that facilitates and enhances various DNA-dependent activities [31]. Recently, the extracellular roles of HMGB has drawn attention of some researchers since Wang et.al [32] firstly reported that HMGB can be released by cultured macrophages and acts as a potential late mediator of lethality due to exotoxin. Many of the discoveries have indicated that HMGB is an important pro-inflammatory cytokine when released from activated innate immune cells or necrotic cells [33]–[35]. It occupies a central role in mediating the local and systemic responses to necrotic cell death and cancer, invasion by pathogens, trauma and sepsis [36]. Antibodies against HMGB have conferred significant protection against some inflammatory diseases in vertebrates such as sepsis, endotoxemia, arthritis and local inflammation in animal models [37], [38], which point to HMGB as a good potential target for therapeutic intervention [35]. Also, HMGB acting as universal sentinels for nucleic-acid-mediated innate immune responses may have implication in the treatment of immunologic disorders [39].

In order to gather knowledge of oyster defense system and find possible ways to prevent or control RLO or LPS (RLO/LPS) induced disease or inflammation, we used sequencing analysis, recombinant protein and antibody preparation to explore the function of Ca-HMGB in the oyster immune system and study its potential function as a pro-inflammatory cytokine by looking at its tissue distribution, response to the challenge of RLO or LPS (RLO/LPS), RLO induced release patterns and variations in subcellular location and function in relation to other cytokines. Furthermore, the ability of anti-CaHMGB (prepared Ca-HMGB’s antibody) to restrain inflammatory reaction and hemocyte apoptosis and necrosis induced by RLO/LPS challenge were also analyzed.

**Figure 1:**
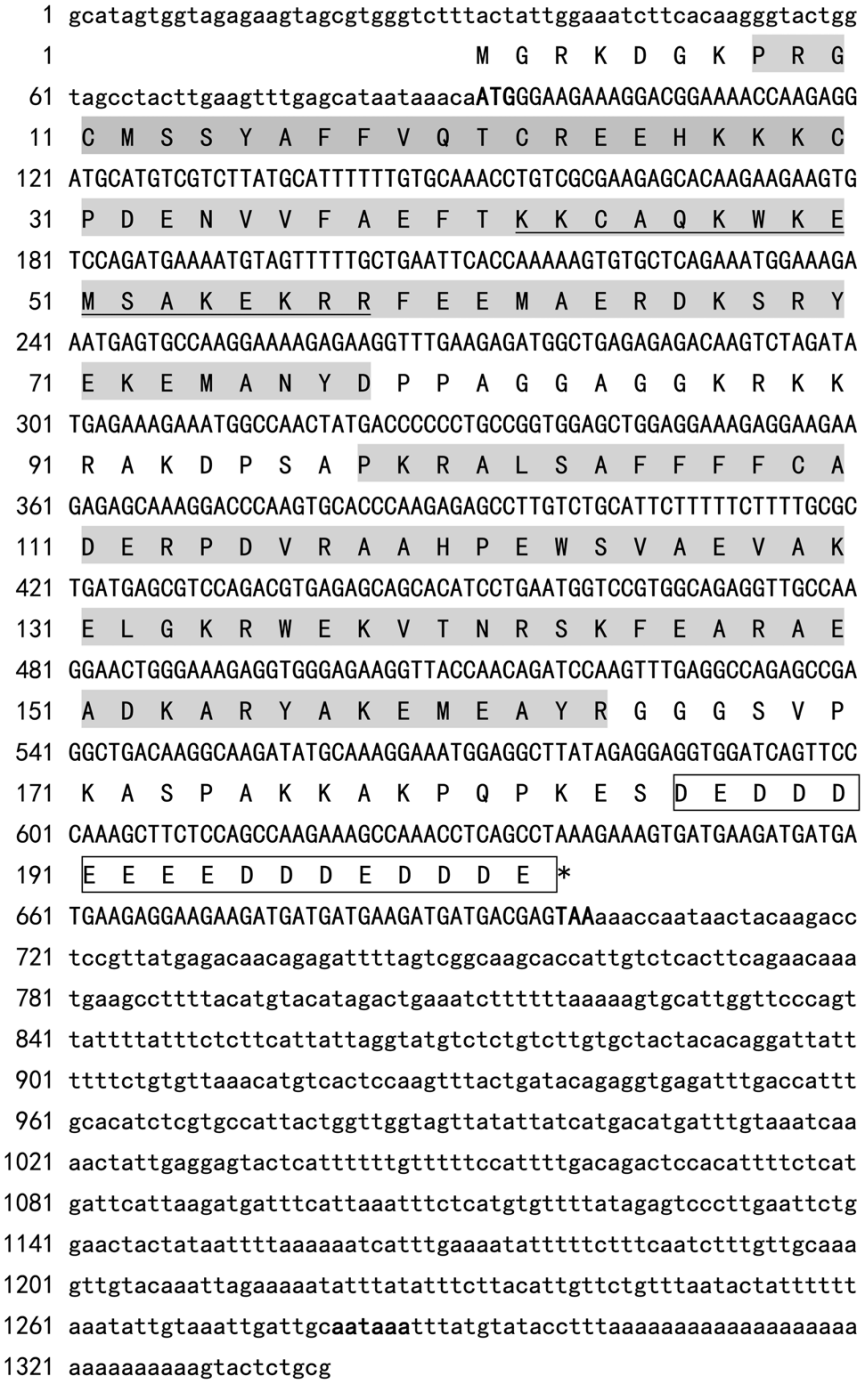
Nucleotide and deduced amino acid sequences of Ca-HMGB from oyster, *Crasstorea ariakensis* Gould. The ORFs of the nucleotide sequences and deduced amino acid are shown in upper-case letters, the 5′ and 3′-UTRs are shown in lower-case letters. Nucleotides and amino acids are numbered on the left of the sequences. The initiation codons, stop codons and the poly (A) signals (aataaa) are in bold. The HMG box A and B motifs are shaded (former one is box A and the later one is box B), the acidic C-tails are boxed and NLS is underlined.

**Figure 2:**
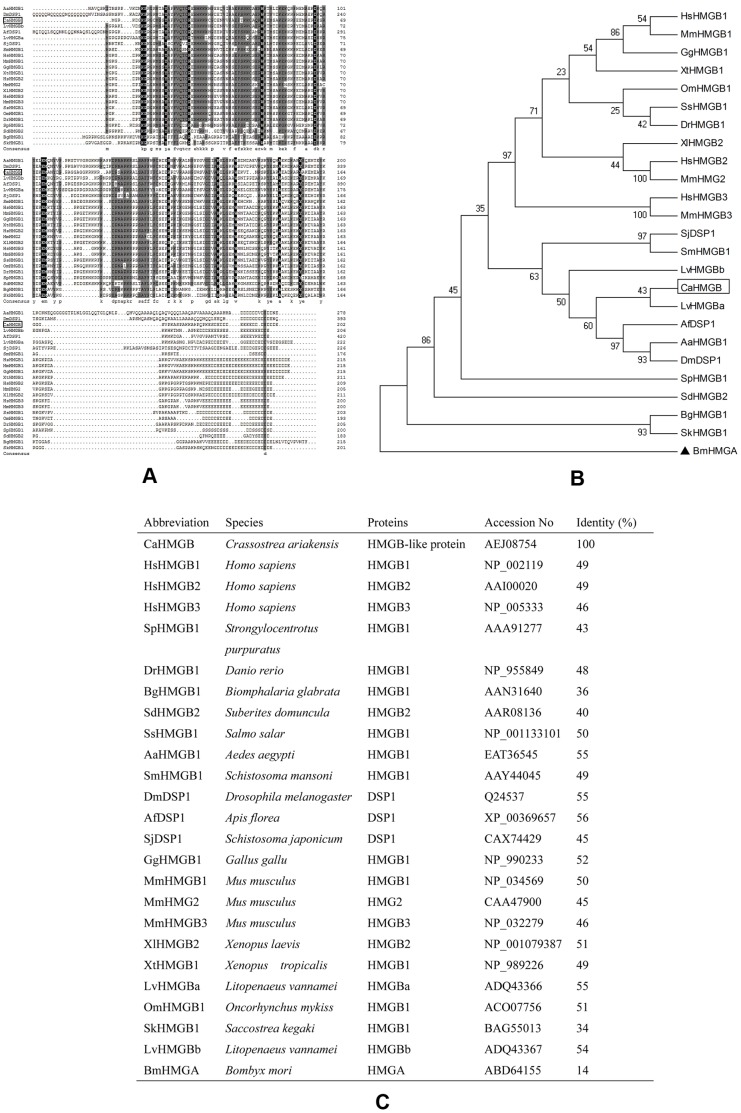
Alignment and phylogenetic analysis of Ca-HMGB amino acid sequences with other animal HMGBs and DSP1s. (A) Multiple alignments. Conserved amino acids were shaded and each shade represents a degree of conservation (black, 100%, grey, 70%). The alignment was taken by ClustalX program. (B) Phylogenetic analysis was performed by MEGA (version 3.1) program. The phylogenetic tree was constructed using neighbor-joining method and bootstrap 1000. The *Bombyx mori* HMGA protein was taken as out-group root. (C) Sequences used in multiple alignments and phylogenic tree analysis together with their identity in amino acid sequences.

## Materials and Methods

### Ethics Statement

The oyster *Crassostrea ariakensis* Gould is one of the most widely consumed seafoods and therefore an economically important marine species in China and around the world. All the experiments were conducted according to the regulations of Chinese local and central governments. The oysters used in this study were bought from the fishermen who cultivated the oysters in Guangxi province. The field studies did not involve endangered or protected species and no specific permits were required in this field study in China.

**Figure 3:**
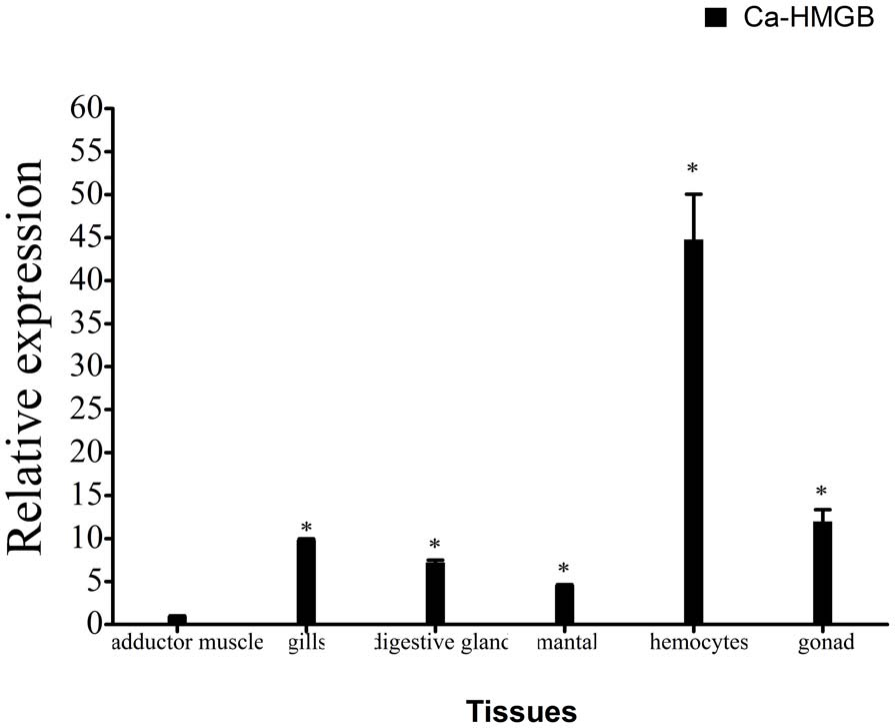
Expression levels of Ca-HMGB were detected in various tissues by real-time RT-PCR. The data were normalized by mRNA expression in the adductor muscle and 28s rDNA was used as endogenous control. The values were presented as mean ± SEM of independent experiments done in triplicates and analyzed by Student’s t-test. *p≤0.05 when compared to control value.

**Figure 4:**
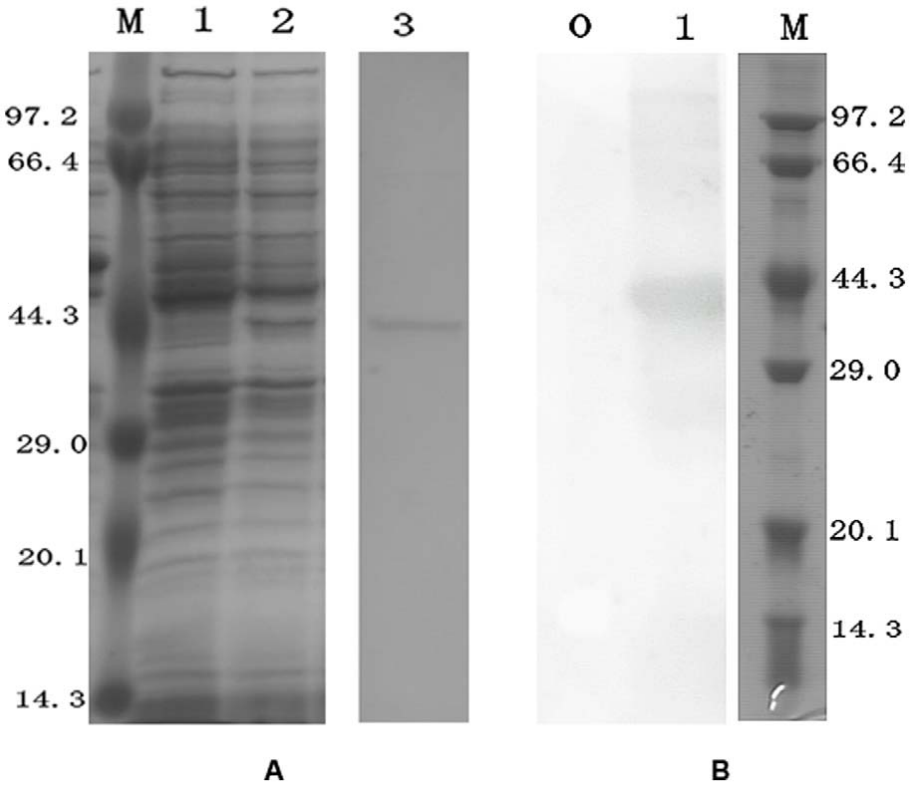
SDS-PAGE and western blotting analysis of Ca-HMGB. (A) SDS-PAGE analysis of pET32a-Ca-HMGB expressed in *E. coli Rossetta* (DE3. M, Protein mark; 1, No induction; 2, 0.5 mM IPTG induction; 3, Purified Ca-HMGB. (B) Western blotting analysis of anti-CaHMGB (1∶5000). 0, pre-immunized rabbit serum; 1, anti-CaHMGB.

### Experimental Oyster and Tissue Isolation

Natural healthy oysters aged 2–3 years old were obtained from Qinzhou bay (Guangxi province, China) and maintained in artificial seawater with a cycling system at 19±1°C for one week before the beginning of the experiment. Three live oysters (untreated) were picked and samples of hemocyte, gills, mantle, digestive glands, gonads and adductor muscle were isolated to identify the tissue-specific expression of Ca-HMGB. Total RNA extraction was performed immediately using the RNA_fast1000_ purification kit (Feijie, China) according to kit procedures. Total RNA isolated from each organ was reverse transcribed into cDNA with M-MLV RTase cDNA Synthesis Kit (Takara, Japan) following the kit instructions and stored at −20°C.

**Figure 5:**
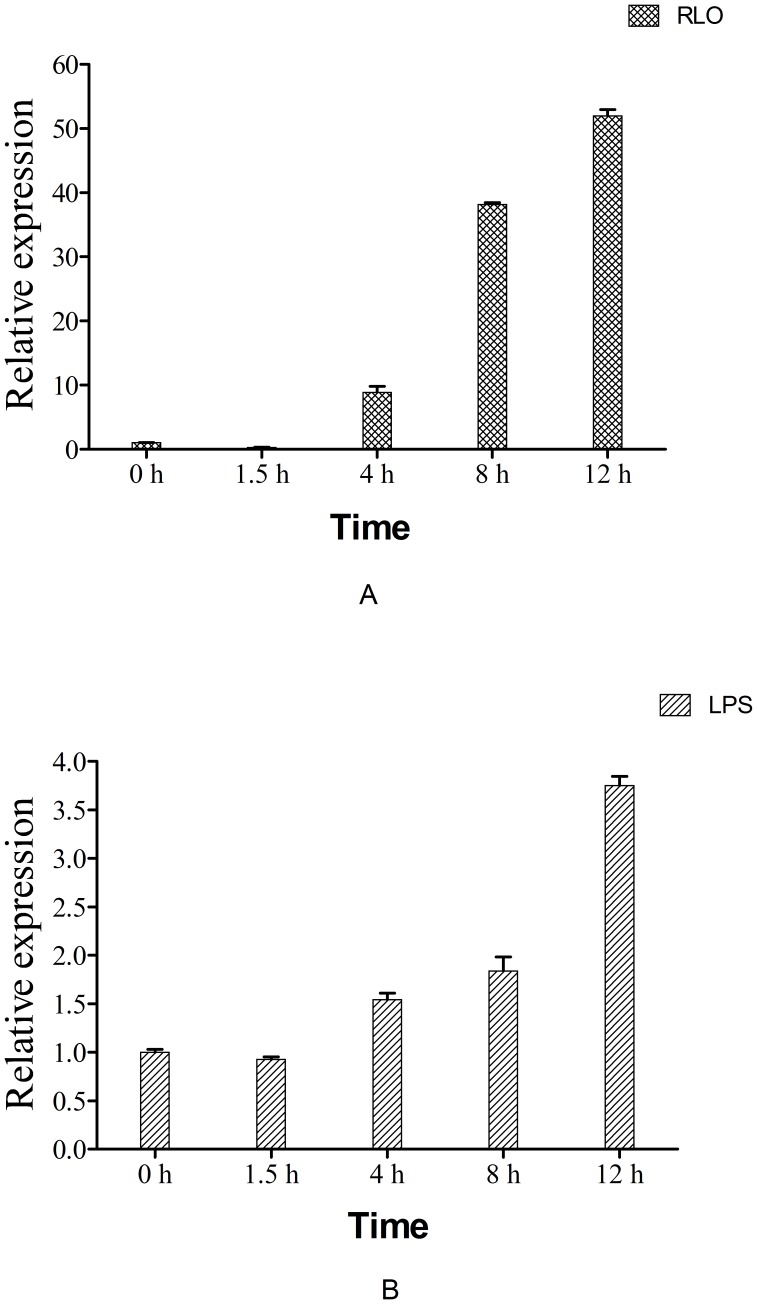
mRNA expression levels of Ca-HMGB in hemocyte monolayers at different times after RLO/LPS incubation. Samples were collected at 0, 1.5, 4, 8 and 12 h after RLO/LPS incubation. Expression levels were determined by real-time RT-PCR. The seawater-incubated groups were used as controls. Expression levels were assessed using 28SrDNA gene for normalization. *p≤0.05 when compared to control value. (A) RLO(1ul/10^6^ cells) incubation; (B) LPS (100 ng/ml)incubation.

**Figure 6:**
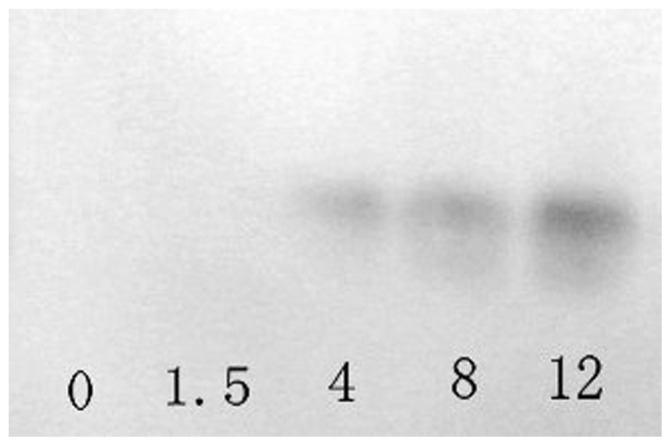
Western-blot analyses of Ca-HMGB in the supernatant of hemocyte monolayers. Samples were collected at 0, 1.5, 4, 8 and 12 h after RLO (1ul/10^6^ cells) incubation.

**Figure 7:**
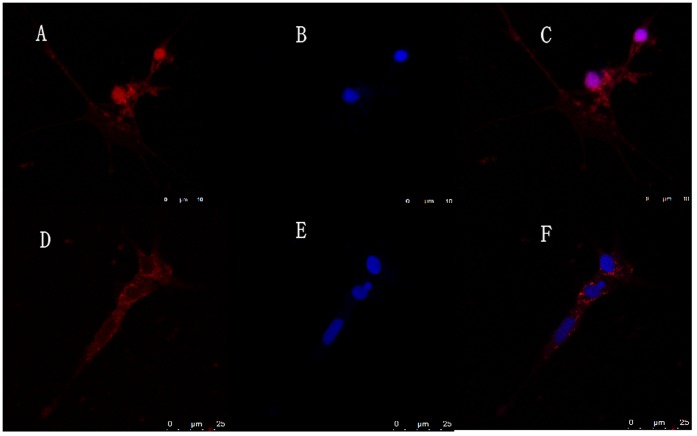
The subcellular localization of Ca-HMGB. A–C: Before RLO challenge. D–F: After RLO challenge. A, D: Immunostaing hemocyte with anti-HMGB; B, E: Imaging of nuclei using the nucleic acid stain DAPI; C, F: Merged.

### RLO Preparation

RLOs were prepared as reported previously [29]. Moribund oysters sampled from Qinzhou bay (Guangxi province, China) were washed with PBS (phosphate-buffered saline, 53.9 mM Na_2_HPO_4_, 12.8 mM KH_2_PO_4_, 2.61 mM NaCl, pH 7.4), and the isolation and purification of RLOs were carried out following the ‘differential speed centrifugation and renografin density gradient centrifugation’ method established by Li and Wu [40]. After that, purified RLOs were cultured using the ‘chick embryo culture’ method (unpublished data), then were collected and centrifuged at 12,000×g for 20 min, and finally resuspended in sterile seawater (OD_600_ = 1.151).

**Figure 8:**
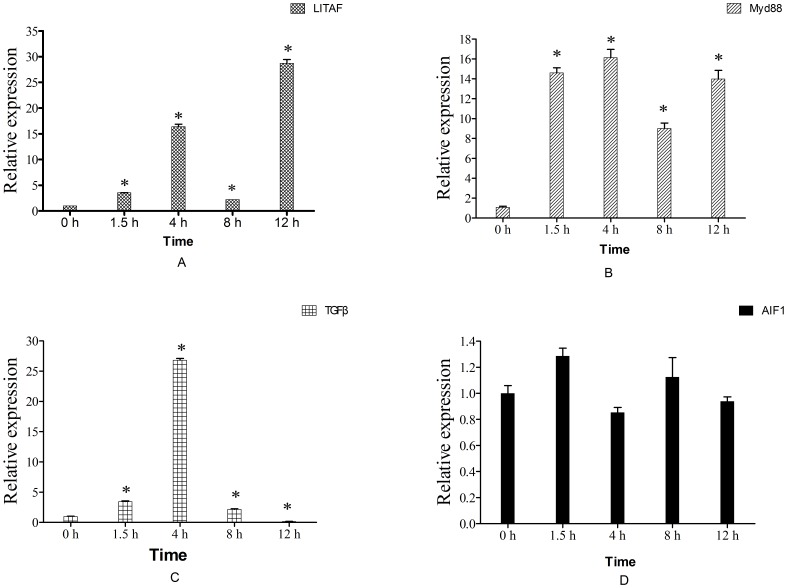
mRNA expression levels of LITAF, Myd88, TGFβ and AIF-1 in hemocyte monolayers. Samples were collected at 0, 1.5, 4, 8 and 12 h after Ca-HMGB protein (1 ug/ml) incubation. Expression levels were assessed by real-time RT-PCR and seawater-incubated groups were used as controls. Expression levels were assessed using 28SrDNA gene for normalization. *p≤0.05 when compared to control value. (A) LITAF; (B) Myd88; (C) TGFβ; (D) AIF-1.

**Figure 9:**
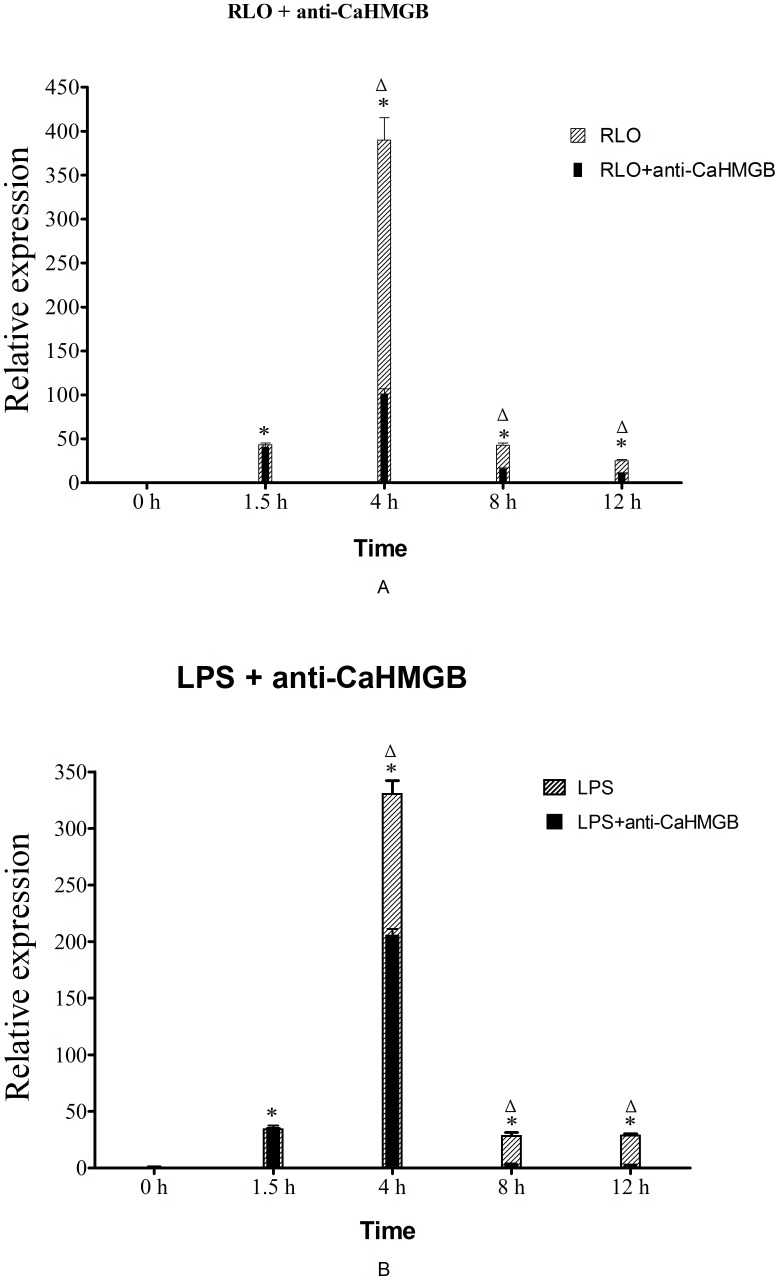
mRNA expression levels of LITAF in hemocyte monolayers. Samples were collected at 0, 1.5, 4, 8 and 12 h after RLO (1ul/10^6^ cells) + anti-CaHMGB (1∶1000) and LPS (100 ng/ml) + anti-CaHMGB (1∶1000) incubation. Expression levels were assessed by real-time RT-PCR and seawater-incubated groups were used as controls. Expression levels were assessed using 28SrDNA gene for normalization. *p≤0.05 when compared to control value. Δ p≤0.05 when RLO/LPS+anti-CaHMGB group value compared to RLO/LPS group value. (A) RLO+ anti-CaHMGB; (B) LPS+anti-CaHMGB.

**Figure 10:**
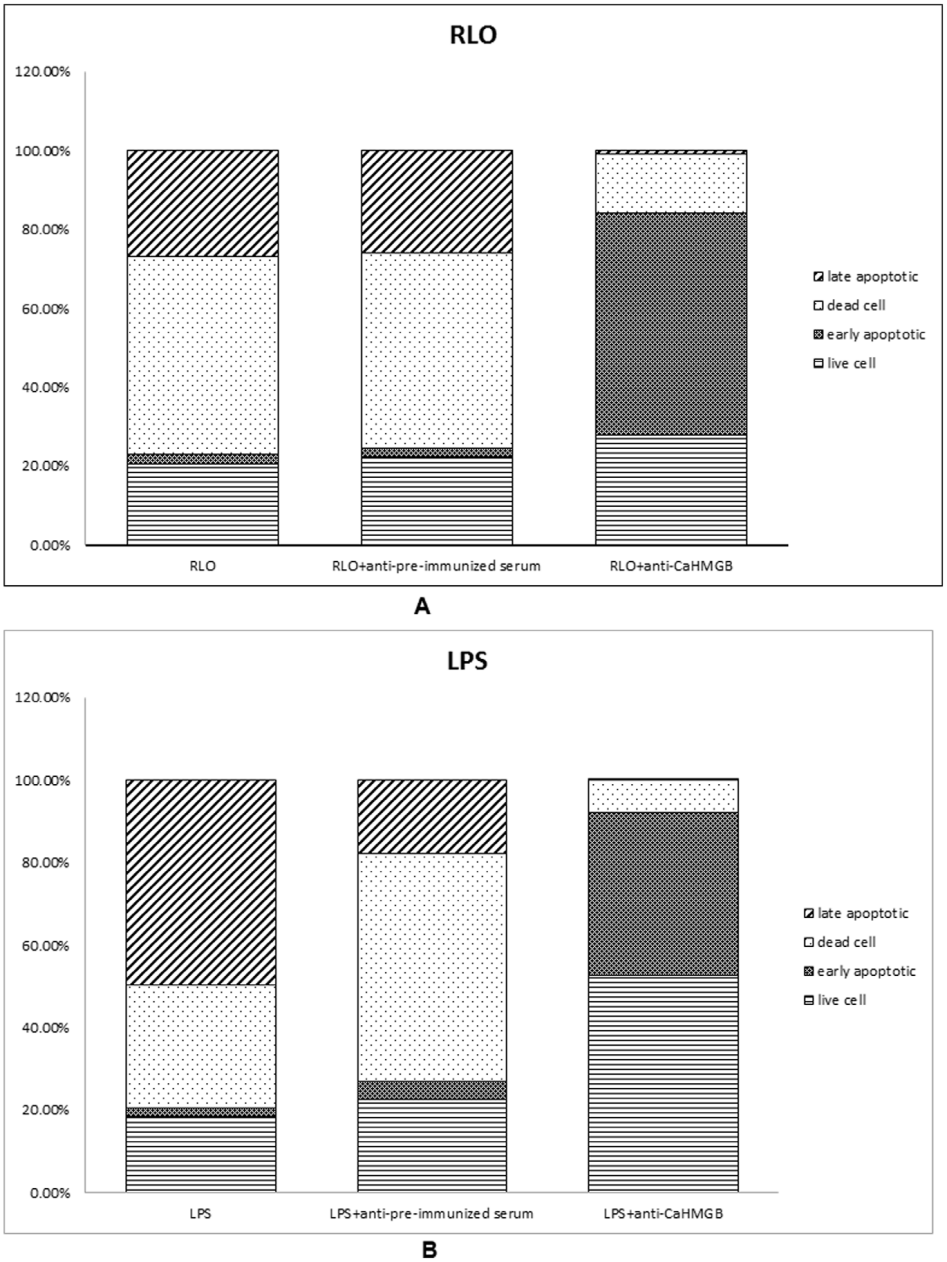
Flow cytometer analyses of hemocyte apoptosis and necrosis. Samples were collected 0 and 12 h after RLO/LPS (RLO, 1ul/10^6^ cells; LPS, 100 ng/ml) + anti-CaHMGB (1∶1000) incubation. The controls were hemocyte incubated with RLO/LPS and RLO/LPS+pre- immunized serum (1∶1000). (A) RLO challenged (B); LPS challenged.

### Sequence Characterization and Phylogenetic Analysis of Ca-HMGB

The RLO-challenged oyster hemocyte cDNA library was constructed previously [29]. Ca-HMGB that contains full open reading frame (ORF) was identified from this library. Homology search was carried out using the BLAST program against the GenBank database at NCBI (http://www.ncbi.nlm.nih.gov/BLAST). ORF was acquired with ORF Finder tool (http://www.ncbi.nlm.nih.gov/gorf/). Domains/motifs were identified using the PROSITE profile data base (http://cn.expasy.org/prosite). Multiple sequence alignment was performed using ClustalW program version 1.8. A phylogenetic tree was constructed using the neighbor-joining method with MEGA 3.1 package and the reliability of the tree was estimated via bootstrap analysis with 1000 replicates.

### Protein Expression, Purification and Polyclonal Antibody Production

Based on the full sequence of Ca-HMGB ORF, specific PCR primers were designed with restriction enzyme (*BamHI* and *XhoI*) sites added to the gene promoter and terminator (Table 1). PCR was carried out using transcribed cDNA from hemocytes as the template and performed under the following conditions: initial denaturation at 94°C for 5 min; followed by 35 amplification cycles (94°C for 30 sec, 51°C for 30 sec, 72°C for 45 sec) and a final elongation step at 72°C for 10 min. PCR products purified by agarose gel electrophoresis were digested with *BamHI* and *XhoI* (Takara, Japan) and ligated to a PET-32a expression vector (digested with the same restriction enzyme) (Novagen, Germany). The recombinant plasmids (PET-CaHMGB) were identified by sequencing and then transformed into competent *E. coli Rossetta* (DE3) cells (Novagen, Germany). The cells were cultured in a shaken incubator at 230 rpm, 37°C in the presence of ampicillin (100 ug/ml) to OD_600_ values of 0.4∼0.6 and then were induced by 0.5 mM isopropyl-1-thio-β-D-galatopyranoside (IPTG, Sigma, USA) at 230 rpm, 30°C for 6 h. Then the cells were collected by centrifugation at 10,000×g for 10 min and the recombinant fusion proteins were purified by affinity chromatography using His•Bind® Purification Kit (Novagen, Germany) following the manufacturer’s protocol. Briefly, the cell pellets were resuspended in ice-cold 1*binding buffer (0.5 M NaCl, 20 mM Tris-HCl, 5 mM imidazole, pH 7.9) and sonicated in tubes on ice. Then the lysate was centrifuged at 14,000 ×g for 20 min and the supernatant was filtered through 0.45 um filters and loaded onto the Ni^2+^-charged and equilibrated His•Bind resin column. The column was then washed with 1*Binding Buffer and 1*Wash Buffer (0.5 M NaCl, 60 mM imidazole, 20 mM Tris-HCl, pH 7.9), and the recombinant fusion proteins were eluted with 1*Elute Buffer (1 M imidazole, 0.5 M NaCl, 20 mM Tris-HCl, pH 7.9) and the purity was checked by 12% SDS-PAGE. Ca-HMGB polyclonal antibody was prepared as discussed previously [12]. In brief, purified proteins were homogenized in complete Freund’s adjuvant (Sigma, USA) and two New Zealand White rabbits were immunized (2 mg purified proteins for each rabbit) three times at 2-week interval. A booster injection was given after another week with 1 mg of purified proteins in incomplete Freund’s adjuvant. Rabbit serum was collected ten days after the last immunization and stored at –80°C.

### Hemocyte Monolayer Preparation and Challenge

Hemocyte monolayers were prepared as described previously [12], [29] and mainly reference Lacoste et al. [41] and Canesi et al. [42]. Briefly, hemolymph was extracted from the pericardial cavity of ten oysters for each experiment, pooled to about 15 ml samples and adjusted to about 10^6^ cells/ml by addition of Hank’s balanced salt solution (HBSS, adjusted to ambient seawater salinity). Hemolymph serum was obtained by centrifuging at 500×g for 5 min and the supernatant was sterilized through a 0.22 um filter. 1 ml of hemolymph was dispensed into sterile Petri dishes and incubated at 15°C for 30 min. Non-adherent hemocytes were carefully rinsed with HBSS, and 1 ml of above filtered hemolymph serum containing penicillin G (50 units/ml) and streptomycin (50 ug/ml) was added to the hemocyte monolayer and kept at 15°C before use.

24 h after the hemocyte monolayers were seeded, we changed the hemolymph serum with fresh serum also containing penicillin G and streptomycin. Then the hemocyte monolayer was treated as follows: (1) hemocytes were incubated with RLOs prepared as before (RLO OD_600_ = 1.151, 1 ul/10^6^ cells) for different periods of time (0 h, 1.5 h, 4 h, 8 h and 12 h); (2) hemocytes were incubated with LPS (100 ng/ml) for different periods of time (0 h, 1.5 h, 4 h, 8 h and 12 h); (3) hemocytes were incubated with purified recombinant Ca-HMGB proteins (1 ug/ml) for different periods of time (0 h, 1.5 h, 4 h, 8 h and 12 h); (4) hemocytes were incubated with RLOs (1 ul/10^6^ cells) and Ca-HMGB polyclonal antisera were produced (1∶1000) for different periods of time (0 h, 1.5 h, 4 h, 8 h and 12 h); (5) hemocytes were incubated with LPS (100 ng/ml) and Ca-HMGB polyclonal antisera were produced (1∶1000) for different periods of time (0 h, 1.5 h, 4 h, 8 h and 12 h); (6) Hemocytes were incubated with filtered seawater. Total RNA from each set of treated hemocytes was then extracted and reverse transcribed as described above.

### Real-time RT-PCR

Real-time RT-PCR was performed to analyze the following: (1) tissue-specific expression of Ca-HMGB; (2) Ca-HMGB mRNA expression profile with RLO/LPS incubation; (3) mRNA expression profile of LITAF (LPS-induced TNFα factor, GenBank No: EU249541), TGFβ (transforming growth factor-beta, Genbank No: EU249542), Myd88 (myeloid differentiation primary response protein 88, GenBank No: EF221769) and AIF1 (allograft inflammatory factor 1, Genbank No: HM749971) with the Ca-HMGB incubation; (4) mRNA expression profile of LITAF with RLO/LPS added/not added with Ca-HMGB polyclonal antiserum incubation.

Gene-specific primers were designed using Primer 5.0 software based on the obtained sequence and reported sequences while the 28 s rDNA gene (GenBank ID: AF137052) was used as an expression standard. The real-time RT-PCR was performed with a SYBR *Premix Ex* Taq™ Kit (Takara, Japan) in an iCycler iQ™ thermocycler (Bio-Rad) using the following procedure: initial denaturation at 95°C for 3 min; followed by 40 cycles of amplification (95°C for 20 sec and 55°C for 40 sec). The gene expression levels relative to the sea water-incubated samples were calculated according to the 2^−ΔΔCT^ method [43], [44]. Experiments were performed in triplicate and the data were presented as the standard errors of the mean (S.E.M.) and were analyzed by Student’s t-test. Differences were considered statistically significant when p values were less than 0.05.

### Western Blotting

Western blot analysis was used to validate the specificity of the prepared Ca-HMGB antibody. Recombinant Ca-HMGB protein was separated on a 12% SDS-PAGE gel and transferred onto a polyvinylidene difluoride (PVDF) membrane (Millipore, USA) using an electrophoretic transfer system (Bio-Rad, USA). Then the membranes were blocked with PBST (PBS pH 7.4, containing 0.1% Tween-20) containing 5% skim milk for 2 h at room temperature, and probed with prepared Ca-HMGB antibody or pre-immunized rabbit serum (1∶5000 diluted in blocking buffer) for 1 h at room temperature. After washing three times with PBST, membranes were incubated with horseradish peroxidase (HRP)-conjugated sheep anti-rabbit IgG antibody (Dingguo, China, 1∶2000 diluted in blocking buffer) for 1 h at room temperature. The immune complexes were detected with an HRP-DAB Detection Kit (Tiangen, China).

Western blot analysis was also used to detect the Ca-HMGB protein levels in the hemolymph serum of cultured hemocyte monolayer when incubated with RLO at different time periods (0 h, 1.5 h, 4 h, 8 h and 12 h). Hemocytes were collected and centrifuged at 500×g for 5 min, and the supernatant was centrifuged a second time at 5,000×g for 5 min. Then the second supernatant was run on a 12% SDS-PAGE gel followed by western blot analysis as described above.

### Immunofluorescence Subcellular Location of Ca-HMGB Protein before and after RLO Incubation

In order to better understand the regulation and function of Ca-HMGB, indirect immunofluorescence technology was used to determine the subcellular location of Ca-HMGB in the hemocytes before and after RLO incubation according to the method described by Brockton et al. [45] with some modification. Briefly, hemocytes (namely hemocyte monolayers with RLO incubation at 0 h and 12 h) were collected by centrifuging at 500×g for 5 min, and leaving a little of the supernatant to resuspend the hemocytes. The suspensions were mounted onto slides and incubated in a humid chamber at room temperature for 15 min. Slides were washed with simple salt solution (SSS, 102.4 g/l NaCl, 1.8 g/l KCl, 5.1 g/l CaCl_2_, 11.8 g/l MgCl_2_, 16.7 g/l MgSO_4,_ and 9 mM EDTA). The hemocytes were fixed as follows: (1) 0.002% gluteraldehyde in SSS for 10 min; (2) 1% formaldehyde and 0.25% Triton X-100 (diluted in 0.15 M PBS, pH 7.4) mixture for 10 min; (3) cold (–20°C) methanol for 15 min. After washing three times in 0.15 mM PBS (5 min each), the slides were incubated in blocking solution (2% v/v normal goat serum) for 30 min in a humid chamber. Then fixed hemocytes were probed with prepared Ca-HMGB antibody (1∶100 diluted in 0.1 M PBS, pH 7.4) for 1 h at room temperature in a humid chamber and then washed. Fixed hemocytes were then incubated with goat anti-rabbit immunoglobulins conjugated to Dye Light 549 (Pierce, 1∶500) and then washed. Next, the hemocytes were stained with 1 ug/ml 4,6-diamidino-2-phenylindole (DAPI) for 5 min and washed again. Lastly, the slides were mounted with 50% glycerol (diluter in 0.5 mol/l Carbonate Buffer solution, pH 9.0), and observed using a TCS SP5 confocal laser scanning system (Leica, Germany) employing helium neon and argon lasers for the appropriate excitation wavelengths. Pre-immune serum was used as negative control.

### Flow Cytometry Analysis

The hemocyte monolayer was cultured as described above. 24 h after seeding, we changed the hemolymph serum (containing penicillin G and streptomycin) and replaced it with fresh hemolymph serum which also contained antibiotics. Then the hemocyte monolayer was treated as follows: (1) hemocytes were incubated with RLO/LPS (RLO: 1 ul/10^6^ cells; LPS: 100 ng/ml); (2) hemocytes were incubated with RLO/LPS and Ca-HMGB polyclonal antiserum (1∶1000); (3) hemocytes were incubated with RLO/LPS and pre-immune serum (1∶1000). 12 h after treatment, flow cytometry analysis was performed to detect the apoptotic cells and necrotic cells using an Annexin V/PI apoptosis kit (MultiSciences Biotech, China). According to the kit instruction, hemocytes (5*10^5^ cells) were collected by centrifuging 500×g for 15 min, resuspended in Annexin-binding buffer with subsequent addition of Annexin V-FITC and Propidum Iodide (PI), and then incubated in the dark for 15 min at room temperature. The data were then analyzed using FACSCalibur (Becton Dickinson, USA) with CellQuest Software (Becton Dickinson). FITC and PI-fluorescence were collected to distinguish among living cells, early apoptotic cell, late apoptotic cell and necrotic cells.

## Results

### Sequence Analysis of Ca-HMGB

A 1340 bp cDNA sequence (GenBank No.HM749973) was obtained by screening the oyster cDNA library we constructed previously [29]. It contains an ORF of 609 bp encoding 203 amino acid residues with a molecular weight of 23.15 kD and a theoretical isoelectric point (PI) of 7.098. Blastp analysis of the deduced amino acid revealed that it is a homologue of HMGB protein similar to that identified in other species. We named it Ca-HMGB. The 5′ and 3′ untranslated regions (UTR) contain 91 bp and 640 bp, respectively. A single typical polyadenylation signal (AATAAA) was found in the 3′ UTR.

Analysis of the deduced amino acid sequence of Ca-HMGB with the pattern database at Prosite (http://prosite.expasy.org/) revealed that it contains two conserved HMG-box (A box and B box) DNA-binding domains. The A box domain corresponded to the 8^th^ to 78^th^ amino acid residues and the B box domain corresponded to the 98^th^ to 164^th^ amino acid residues. The two domains shared 34% identity in deduced amino acid sequence. The acidic C-tail found in the Ca-HMGB contains 17 contiguous aspartic or glutamic residues and a bipartite nuclear localization signal NLS_BP was also identified from the 42^nd^ to 58^th^ amino acid residues. Additionally, several biologically active site motifs were also identified from Ca-HMGB including three amidation sites (residues 1^st^ –4^th^, 85^th^–88^th^ and 132^nd^ –135^th^), two N-myristoylation sites (residues 10^th^–15th and 82^nd^–87^th^), one N-glycosylation site (residues 141^st^–144^th^), four protein kinase C phosphorylation sites (residues 21^st^–23^rd^, 41^st^–43^rd^, 52^nd^–54^th^ and 140^th^ –142^nd^ ), six casein kinase II phosphorylation sites (residues 21^st^–24^th^, 52^nd^–55^th^, 68^th^ –71^st^, 124^th^–127^th^, 143^rd^–146^th^ and 185–188^th^ ) and three tyrosine kinase phosphorylation sites (residues 69^th^–77^th^, 148^th^–156^th^ and 155^th^–163^rd^) (Figure 1).

Multiple alignment of Ca-HMGB with HMGB-related proteins from various animals (including invertebrates and vertebrates) showed sequence identity ranging from 34%−56% (Figure 2A). A phylogenetic tree was generated based on multiple alignment using the neighbor-joining method with the *Bombyx mori* HMGA protein used as the root. The result showed that Ca-HMGB had the highest homology to *Litopenaeus vannamei* HMGBa and the lowest homology to other mollusk HMGBs (Figure 2B). The sequences used for multiple alignment and phylogenic analysis and their percentage identity with Ca-HMGB were shown in Figure 2C.

### Tissue-specific Expression of Ca-HMGB

The expression levels of Ca-HMGB in various tissues including hemocyte, gills, mantle, digestive glands, gonads and adductor muscle were investigated using real-time RT-PCR. The designed primer pairs were verified in their specificity by sequencing the PCR product before use. The results showed that Ca-HMGB was ubiquitously expressed in all examined tissues and the expression level in hemocyte is at least 4-fold higher compared to the others (Figure 3).

### Protein Expression, Purification and Polyclonal Antibody Production

The complete ORF of Ca-HMGB was cloned into the PET32a expression vector. A protein with a molecular weight of about 41 kD was expressed in *E. coli Rossetta* (DE3) and purified using the Ni^2+^-charged affinity columns. The purified protein showed the expected molecular weight (23.15 kD Ca-HMGB+about 18 kD PET32a tags) as detected by SDS-PAGE and was further confirmed by western bolt analysis using prepared anti-CaHMGB rabbit anti-serum (Figure 4A, B).

### Expression Profile of Ca-HMGB in the Hemocyte Monolayer after RLO/LPS Challenge

The hemocyte monolayer was prepared and the real-time RT-PCR approach was used to analyze the mRNA expression profile of Ca-HMGB when challenged with RLO/LPS. For the RLO challenge, the mRNA expression level of Ca-HMGB significantly decreased at first (about 29% of the control group at 1.5 h post-challenge), then the expression levels significantly increased continuously at 4–12 h post-challenge (about 9, 38 and 52 times compared to control groups at 4 h, 8 h and 12 h, respectively) (Figure 5A). For the LPS challenge, the mRNA expression level of Ca-HMGB was not statistically different (p value >0.05) at 1.5 h post-challenge, however the expression levels significantly increased continuously at 4–12 h post-challenge (about 1.5, 1.8 and 3.8 times compared to control group at 4 h, 8 h and 12 h, respectively) (Figure 5B).

### Ca-HMGB Protein Profile in the Supernatant of Hemocyte Monolayer after RLO Challenge

Western blot analysis showed that there was no obvious Ca-HMGB protein detected in the supernatant at 0 h and 1.5 h post-challenge. However the Ca-HMGB protein levels increased continuously from 4 h to 12 h post-challenge (Figure 6).

### Subcellular Location of Ca-HMGB Protein in the Hemocyte before and after RLO Challenge

To investigate the subcellular localization of Ca-HMGB protein, we performed indirect immunofluorescence labeling using the anti-CaHMGB antibody (Figure 7A–F). Confocal microscopy observation results showed that Ca-HMGB was located both in the cell nucleus and cytoplasm before RLO challenge (Figure 7A–C). And 12 h after RLO challenge, Ca-HMGB was mainly located in the cytoplasm (Figure 7D–F).

### The effects of Ca-HMGB on Expression Levels of LITAF, TGFβ, Myd88 and AIF1 in Oyster Hemocyte

Real-time RT-PCR analysis was used to investigate the effects of Ca-HMGB incubation *in vitro* on mRNA expression levels of immune-related genes LITAF, TGFβ, Myd88 and AIF1. The results showed that Ca-HMGB could up-regulate the expression levels of LITAF, Myd88 and TGFβ but had no obviously function in regard to AIF1 (p value >0.05) (Figure 8 A–D).

The mRNA expression level of LITAF increased immediately after Ca-HMGB incubation *in vitro* and occurred in a biphasic pattern (Figure 8A). The early peak occurred at 4 h post-incubation with the expression level about 16.4 times higher compared to the control group. The later peak occurred at 12 h post-incubation with the expression level about 28.7 times higher compared to the control group. And the expression levels at 1.5 h and 8 h post- incubation are about 3.5 and 2.2 times higher compared to the control group, respectively. Similar biphasic pattern occurred in the mRNA expression of Myd88 when incubated with Ca-HMGB protein. The early peak occurred at 4 h post-incubation and is about 14.8 times higher compared to the control group, while the later peak occurred at 12 h post-incubation and is about 12.8 times higher compared to the control group. Aside from the biphasic pattern the expression levels of Myd88 at 1.5 h and 8 h post-incubation are about 13.4 and 8.28 times higher compared to control group (Figure 8B). The mRNA expression level of TGFβ also increased immediately when incubated with Ca-HMGB, but had different kinetics patterns. It had only one peak at 4 h post-incubation and is about 26.9 times higher compared to the control group. The expression levels at 1.5 h and 8 h post-incubation were also up-regulated to about 3.5 and 2.2 times higher respectively compared to the control group (Figure 8C).

### The effect of Anti-CaHMGB on the Reduction of RLO/LPS-induced Increase of LITAF’s mRNA Expression Level

In order to investigate the effect of the prepared Ca-HMGB antibody on decreasing inflammatory reactions *in vitro*, we added prepared anti-CaHMGB polyclonal antiserum to the RLO/LPS challenged hemocyte monolayer, and then performed real-time RT-PCR to analyze the mRNA expression level variation of LITAF at 1.5 h, 4 h, 8 h and 12 h post-treatment. The results showed that the RLO/LPS challenge could up-regulate the expression level of LITAF in the hemocyte, while administration of anti-CaHMGB can reduce RLO/LPS-induced up-regulation of LITAF. The reduction appeared from 4–12 h post-treatment (Figure 9).

When challenged with RLO, mRNA expression levels of LITAF experienced up-regulation immediately and are about 43.32, 390.18, 42.95 and 25.27 times higher compared to the control group at 1.5 h, 4 h, 8 h and 12 h post-challenge, respectively. Administration of anti-CaHMGB antibody significantly reduced the expression levels of LITAF to 101.41, 16.08 and 11.06 times higher compared to the control group at 4 h, 8 h and 12 h post-challenge, respectively, with the reduction rates around 74%, 62.57% and 56.23%, respectively (Figure 9A). Howerver the reduction is not significant at 1.5 h post-challenge (p value >0.05) and about 40.93 times higher compared to the control group.

When challenged with LPS, mRNA expression levels of LITAF experienced up-regulation immediately and are about 35.59, 330.81, 28.73 and 29.14 times higher compared to the control group at 1.5 h, 4 h, 8 h and 12 h post-challenge, respectively. Administration of anti-CaHMGB significantly reduced the expression levels of LITAF to 206.06, 28.73 and 29.14 times higher compared to the control group at 4 h, 8 h and 12 h post-challenge, respectively, with reduction rates around 37.71%, 89.99% and 86.62%, respectively (Figure 9B). Howerver the reduction is not significant at 1.5 h post-challenge (p value >0.05) and about 34.73 times higher compared to the control group.

### The Effect of Anti-CaHMGB Antibody on the Reduction of RLO/LPS-induced Hemocyte Apoptosis and Necrosis Rates

In this study, flow cytometry analysis was used to detect the apoptosis and necrosis rates of hemocytes *in vitro* to further analyze anti-CaHMGB antiserum function. The results showed that administration of anti-CaHMGB can reduce the RLO/LPS-induced hemocyte cell apoptosis and necrosis rates and increase the cell survival rate.

Compared to the two control groups (RLO challenged group and RLO+pre-immune serum challenged group), the percentage of late apoptotic cells in the RLO+anti-CaHMGB antiserum (1∶1000) challenged group both decreased by 26%, the percentage of necrotic cells both decreased by 35%, the percentage of early apoptotic cells both increased by 54%, and the percentage of surviving cells increased by 7% and 5%, respectively (Figure 10A).

Compared to the two control groups (LPS challenged group and LPS+pre-immune serum challenged group), the percentage of late apoptotic cells in the LPS+anti-CaHMGB antiserum (1∶1000) challenged group were decreased by 50% and 18% respectively, the percentage of necrotic cells were decreased by 22% and 48% respectively, the percentage of early apoptotic cells were increased by 37% and 35% respectively, and the percentage of surviving cells were increased by 34% and 30% respectively (Figure 10B).

## Discussion

In mammals, there are three members of HMGB proteins, namely HMGB1, HMGB2 and HMGB3 [46]. All of them are characterized by three conserved domains, namely two HMG boxes (A and B) and the acidic C-tail. The three HMGB members share more than 80% sequence identity in amino acid sequence [31]. In addition, dorsal switch protein 1 (Dsp1) is the only invertebrate orthologue of mammalian HMGB proteins and it contains a N-terminal Q-rich tail in addition to the three conserved domains mentioned above [47]. In our study, Ca-HMGB was identified containing all the three characteristic HMGB domains but without the characteristic Dsp1 Q-rich tail domain. It is homologous to vertebrate HMGBs. Multiple sequence alignment and a constructed phylogenetic tree showed that HMGBs are conserved in amino acid sequence, especially at the N-terminal and Ca-HMGB had higher identity to the arthropoda HMGB and DSP1 rather than the used mollusk HMGBs [48].

Tissue-specific expression revealed that Ca-HMGB mRNA showed maximal expression levels in hemocytes, which is thought to play a key role in invertebrate innate immunity [3], [49], [50]. This result indicates that Ca-HMGB might take part in the oyster immune system and is similar to the HMGBa from *Litopenaeus vannamei,* which shows maximal expression level in hepatopancreas, the main immune organ of shrimp [51].

Mammalian HMGB can be released from activated innate immune cells or necrotic cells and function as proinflammatory cytokines [33]–[35]. When stimulated with LPS or some proinflammatory cytokines like TNF, IL-1β and IFN-γ, HMGB can be released from activated innate immune cells like macrophages and monocytes at more than 8 h and reaches a plateau in expression levels at around 18–24 h [32], [52], [53]. In addition, the necrotic or damaged cells also passively release HMGB protein into extracellular medium [56], [57]. Since HMGB lacks a signal peptide, its secretion is thought to be via a nonclassical, vesicle-mediated secretory pathway and HMGB protein is firstly translocated from nuclear to cytoplasm before release [54], [55]. In order to demonstrate the release pattern of Ca-HMGB in this study, we used the pathogen RLO to stimulate the prepared oyster hemocyte monolayer, and western blots were used to detect the release of Ca-HMGB and indirect immunofluorescence labeling was used to determine the subcellular translocation of Ca-HMGB in the hemocytes before and after RLO stimulation. Western blot analysis results showed that 4 h after RLO stimulation, Ca-HMGB was released from hemocyte cells and the protein levels in the extracellular medium increased continuously in the detection period (0–12 h). Confocal microscopy results of the indirect immunofluorescence labeling showed that before challenge, Ca-HMGB localized both in the cell nucleus and cytoplasm while 12 h after RLO challenge, Ca-HMGB mainly localized to the cytoplasm. These results showed that Ca-HMGB can be released extracellularly to take part in the inflammatory reaction and its subcellular localization varies when stimulated with RLO.

In addition, real-time RT-PCR analyses were taken to detect the variations in Ca-HMGB mRNA expression levels when stimulated with RLO/LPS in this study. The results showed that the expression levels of Ca-HMGB increases continuously from 4–12 h post-challenge with RLO/LPS, indicating that Ca-HMGB can take part in the oyster defense system against RLO/Gram^−^ bacterial infection. The difference between RLO and LPS challenge was at 1.5 h post-challenge. For RLO, expression level is down-regulated and for LPS, no significantly change was observed. Similar results were observed in LvHMGBa and LvHMGBb, where the expression levels were significantly increased at 12 h after stimulated with WSSV and reached a peak at 24 h [51].

Once released, mammalian HMGB can stimulate the production of pro-inflammatory cytokines (eg., TNF, IL-1α, IL-1β, IL-6, IL-8, MIP-1α, MIP-1β) as well as increase nuclear translocation of NF-κB and mediate a variety of inflammatory responses [32], [56], [57]. Compared with LPS stimulation, recombinant HMGB stimulation causes a biphasic and delayed TNF and IL-6 synthesis in human monocytes in a dose dependent manner [56]. HMGB activated TNF synthesis occurred in a biphasic pattern and activated a significantly higher number of TNF-producing monocytes during the later peak. Thus HMGB was suggested to be a monocyte-activating cytokine [56]. In addition, TLR2 and TLR4 are probably the receptors responsible for HMGB-mediated production of pro-inflammatory cytokines. Their antibodies attenuate HMGB-induced pro-inflammatory cytokine release in a dose dependent manner and HMGB activates significantly less TNF release from Myd88 and TLR4 knockout mice [58]. Similarly, SmHMGB from *Schistosoma mansoni* is a potent inducer of pro-inflammatory cytokines such as TNFα, IL-1Rα, IL-2Rα, IL-6, IL-13, IL-13R, IL-15 and MIP-1α from mouse peritoneal macrophages and was thought to be a key molecule in the development of host inflammatory immune response associated with schistosomiasis [59].

In this study, we used purified recombinant Ca-HMGB protein to stimulate prepared oyster hemocyte monolayer, and real-time RT-PCR was used to detect the expression variations of LITAF, AIF1, Myd88 and TGFβ at the mRNA level. The results showed that Ca-HMGB can up-regulate the expression levels of LITAF, Myd88 and TGFβ but had no obviously function in relation to AIF1. The LITAF and Myd88 mRNA expression variation occurred in a biphasic pattern while TGFβ expression variation showed only a single peak. However, mRNA expression levels of these three genes increased immediately without delayed. Among the four genes detected, LITAF is an important transcription factor and believed to regulate the expression of TNFα [60], [61]. TNFα and TGFβ are multifunctional cytokines and most commonly examined in disease studies, participating for example in several biological processes such as cell differentiation and inflammatory reaction [62], [63]. Myd88 is the key downstream adapter for most Toll-like receptors (TLRs) and interleukin-1 receptors (IL1Rs) and acts as a negative regulator of IL1R/TLR/MYD88 signals and leads to a controlled negative regulation of innate immune responses [64], [65]. AIF1 is an IFN-γ-inducible cytokine and identified as one of the key genes associated with several kinds of inflammatory response-related diseases [66], [67]. Therefore, our results confirmed previous studies, that is, HMGB has potential pro-inflammatory cytokine functions and might take part in signal transduction through Myd88-mediating pathways.

There have been some reports that administration of specific HMGB antibodies or antagonists can protect mice against lethal endotoxemia, systemic inflammation and sepsis even with delayed treatment [37], [38]. Addition of anti-SmHMGB1 polyclonal antibodies to mouse peritoneal macrophage culture blocked the TNFα-inducing effect of SmHMGB in a concentration dependent manner, suggesting that TNFα-inducing activity of SmHMGB1 is specific [59]. In our study, real-time RT-PCR was performed to observe prepared anti-CaHMGB polyclonal antiserum function on reducing RLO/LPS-induced LITAF expression and flow cytometry was used to analyze anti-CaHMGB function on reducing RLO/LPS-induced hemocyte cell apoptosis and necrosis rates. The results showed that administration of prepared anti-CaHMGB polyclonal antiserum effectively blocked RLO/LPS-induced up-regulation of LITAF and the late apoptosis rate and necrosis rate of RLO/LPS-induced hemocyte cells were also reduced while the early apoptosis rate and living cell rate increased. These results indicate that prepared anti-CaHMGB polyclonal antiserum can significantly inhibit RLO/LPS-induced inflammatory reaction and effectively confer the oyster significant protection against RLO/Gram negative bacterial infection.

The suppression of RLO/LPS-induced up-regulation of LITAF happened 4 h after the administration of the antiserum rather than immediately afterwards. Also, Ca-HMGB can be released extracellularly at 4–12 h after RLO challenge and the administration of Ca-HMGB can up-regulate the expression level of LITAF. In summary, we suggest that Ca-HMGB might be a potential target to prevent and control RLO/LPS-induced disease or inflammation and anti-CaHMGB can confer the oyster significant protection against infection by restraining the pro-inflammatory reaction induced by Ca-HMGB. Further studies regarding the details of this molecular pathway are worthwhile.
