# Correction: HMGB in Mollusk *Crassostrea ariakensis* Gould: Structure, Pro-Inflammatory Cytokine Function Characterization and Anti-Infection Role of Its Antibody

**DOI:** 10.1371/annotation/694da01b-1156-4a32-ae7c-73822eba2ef6

**Published:** 2013-10-16

**Authors:** Ting Xu, Shoubao Yang, Jiasong Xie, Shigen Ye, Ming Luo, Zewen Zhu, Xinzhong Wu

The times listed in the second paragraph are incorrect in the sentence:

"Administration of anti-CaHMGB significantly reduced the expression levels of LITAF to 206.06, 28.73 and 29.14 times higher compared to the control group at 4 h, 8 h and 12 h post-challenge, respectively, with reduction rates around 37.71%, 89.99% and 86.62%, respectively."

The correct times should be: 3.88 and 3.90 at 8 h and 12 h post-challenge. And the reduction rate at 8 h post-challenge should be 86.49% rather than 89.99%. 

